# Compositors and Consumption

**Published:** 1858-10

**Authors:** 


Compositors and Consumption.

The Report on the Sanitary Condition of the Army recently published, proves that our soldiers, but especially the infantry of the line and the foot guards, are subject to a very high mortality, a great part of which is attributed to pulmonary consumption. It is assumed in the Report that this excess of mortality from consumption is traceable, at least in part, to the narrow space allotted to the soldier in the barrack and guard-rooms: but as no proof of the dependence of pulmonary consumption on this cause is given in the Report, Dr. Guy republishes in Beale's *Archives*, from his evidence laid before the Health of Towns Commission in 1844, the following table based upon measurements of the offices of letterpress printers, and the number of compositors working in them, together with the answers to certain simple questions addressed to the men themselves.

| | No. per cent. subject to Spitting Blood. | Catarrh. |
|---|---|---|
| 104 men having less than 500 cubic feet of air to breathe | 12·50 | 12·50 |
| 115 men having from 500 to 600 cubic feet of air to breathe.................................... | 4·35 | 3·48 |
| 101 men having more than 600 cubic feet of air to breathe | 3·96 | 1·98 |

The number of compositors who answered in the affirmative Dr. Guy's question, Had they ever spit blood? corresponded very closely to the number actually suffering under consumption; just as the number stating that they were subject to colds afforded a good indication of the number in the three classes who were predisposed by the same close and confined atmosphere to suffer by exposure to the common causes of diseases of the chest.

